# Early Detection of Myocardial Bioenergetic Deficits: A 9.4 Tesla Complete Non Invasive ^31^P MR Spectroscopy Study in Mice with Muscular Dystrophy

**DOI:** 10.1371/journal.pone.0135000

**Published:** 2015-08-11

**Authors:** Weina Cui, Albert Jang, Pengyuan Zhang, Brian Thompson, DeWayne Townsend, Joseph M. Metzger, Jianyi Zhang

**Affiliations:** 1 Department of Medicine/Cardiology, University of Minnesota Medical School, Minneapolis, Minnesota, United States of America; 2 Department of Integrative Biology & Physiology, University of Minnesota, Minneapolis, Minnesota, United States of America; Georgia Regents University, UNITED STATES

## Abstract

**Background:**

Duchenne muscular dystrophy (DMD) is the most common fatal form of muscular dystrophy characterized by striated muscle wasting and dysfunction. Patients with DMD have a very high incidence of heart failure, which is increasingly the cause of death in DMD patients. We hypothesize that in the in vivo system, the dystrophic cardiac muscle displays bioenergetic deficits prior to any functional or structural deficits. To address this we developed a complete non invasive 31P magnetic resonance spectroscopy (31P MRS) approach to measure myocardial bioenergetics in the heart in vivo.

**Methods and Results:**

Six control and nine mdx mice at 5 months of age were used for the study. A standard 3D -Image Selected In vivo Spectroscopy (3D-ISIS) sequence was used to provide complete gradient controlled three-dimensional localization for heart 31P MRS. These studies demonstrated dystrophic hearts have a significant reduction in PCr/ATP ratio compare to normal (1.59±0.13 vs 2.37±0.25, p<0.05).

**Conclusion:**

Our present study provides the direct evidence of significant cardiac bioenergetic deficits in the in vivo dystrophic mouse. These data suggest that energetic defects precede the development of significant hemodynamic or structural changes. The methods provide a clinically relevant approach to use myocardial energetics as an early marker of disease in the dystrophic heart. The new method in detecting the in vivo bioenergetics abnormality as an early non-invasive marker of emerging dystrophic cardiomyopathy is critical in management of patients with DMD, and optimized therapies aimed at slowing or reversing the cardiomyopathy.

## Introduction

Duchenne Muscular Dystrophy (DMD) is a genetic disorder which is a result of mutations in the DMD gene, and leading to the absence of the cytoskeletal protein dystrophin. DMD is one of the most common muscular dystrophies and affects 1:3,500–1:5,000 boys born in the United States [1]. DMD is characterized by progressive skeletal muscle weakness and premature death [2]. Nearly all DMD patients develop cardiomyopathy by the age of 18, which is progressive and also contributes to premature death in these patients [3]. Dystrophin is a large cytoskeletal protein that is critical in forming a physical connection between the internal cytoskeleton and the extracellular matrix [4, 5]. It has been proposed that dystrophin functions as a mechanical buffer protecting the sarcolemmal membrane from the forces of contraction [6, 7]. Recent studies have demonstrated abnormalities in mitochondrial function may also contribute to the overall disease process [8–10]. These studies raised the possibility that the energetics of the dystrophic heart may be a leading early indicator of disease.

We hypothesize that ^31^P MRS will provide an early detection of myocardial bioenergetic changes of the dystrophic heart. It is further hypothesized that myocardial bioenergetic deficits of the dystrophic heart will be present prior to the development of hemodynamic or structural changes in dystrophic heart. There is an important need within the DMD field to identify reliable and quantitative early biomarkers of muscle disease onset and progression [11]. Such biomarkers are essential for evaluating the efficacy of clinical treatment interventions. The demonstration of in vivo energetics as an early non-invasive physiological marker of emerging dystrophic cardiomyopathy could be critical for the evaluation of therapies aimed at slowing or reversing the cardiomyopathy of DMD.

## Methods

### Subjects

All animal protocols were approved by the University of Minnesota Institutional Animal Care and Use Committee and performed in accordance with the National Research Council’s Guide for the Care and Use of Laboratory Animals. Six C57BL/10 mice (3 Male, 3 Female, weight 28-38g, age 22 to 28 weeks) and nine mdx mice (3 male, 6 female, weight 27 to 31g, age 22 to 28 weeks) were used for the study.

### Animal Preparation

Animals were induced with 2% to 3% isoflurane and a 1:1 mixture of O_2_:N_2_O. Then mice were placed prone and fixed in a custom built mouse holder with a nose cone system, which is connected with a small animal ventilator (Kent scientific, Torrington, CT, USA). During the whole process of MR scanning, heart rate, body temperature and respiration rate were monitored (SA Instruments, Stony Brook, NY, USA). The mice were maintained anesthetized (breathing rate 120 to 150/min) with 0.5% to 1% isoflurane. Body temperature was maintained at about 37 degree with a circulating warm water system and a heating fan.

### Coil design

**Fig 1:**
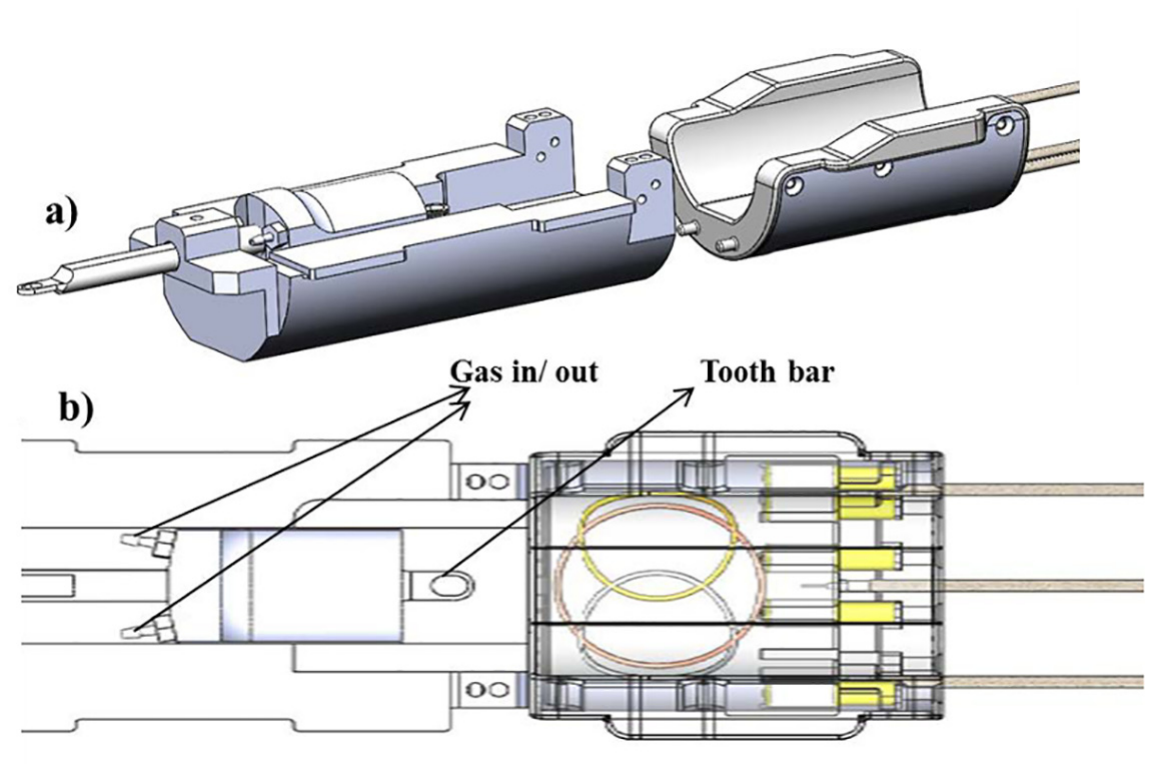
Coil and animal holder system. Fig 1a is the whole picture the of system. Fig 1b brought close details. The tooth bar was used to position the mouse to the nose cone. The in/out outlets allow the stable gas flow of O_2_/N_2_O from the ventilator. The mouse heart was positioned directly on the center of coil.

We have designed, developed, and rigorously tested a new coil for non-invasive follow up studies of mouse model of muscular dystrophy. The coil (Fig 1) consists of a 2 proton transmitter-receiver (TR)-loops (3.5cm diameter) driven in quadrature. A third phosphorous coil (2.5cm diameter) is nested between the 2 proton loops. All coils are built into a custom machined Delrin housing and are manually tuned and matched via variable capacitors. Cable traps are also installed on each RF cable to improve performance. The entire device is designed to fit in the 9.4T magnet with 31 cm bore.

### Magnetic Resonance Spectroscopic Protocol

All MRI studies were performed using a 9.4 T Varian/Magnex (31 cm bore) system. The mouse was positioned precisely on the top of the center of the home built ^1^H/^31^P coil. Three dimension multi slice anatomic ^1^H images were acquired using a tagcine imaging sequence triggered by respiration rate (repetition time TR = 180ms, TE = 3.04ms, flip angle 20 deg, CINE frames = 2, Averages = 2, matrix = 128x128, field of view 40x40mm, slice thickness 2mm). The mouse heart was localized on the 3 dimensional images and used for the following ^31^P MRS study without repositioning the animal. Standard 3D Image Selected In vivo Spectroscopy (3D-ISIS) sequence (was used to provide complete gradient controlled three-dimensional localization for heart ^31^P MRS. The total acquisition time was 50 minutes (TR = 2 or 4 sec, voxel volume is around 300 mm^3^ depending on individual animal, spectral width 10Khz). The free induction decays were zero-filled, and a line broadening of 50 Hz was applied before fast Fourier transformation. The resonance peaks of PCr (Phosphocreatine) and ATPγ were integrated. The ratios of PCr to ATPγ were then corrected based on the global spectra of TR = 2 sec, TR = 4 sec and TR = 12 sec, to compensate for the partial saturation effect.

### ATP determination in mouse hearts

Mouse hearts were flash frozen in liquid nitrogen immediately after extraction. Hearts were crushed while frozen and crushed tissue was weighed for normalization. ATP was extracted from the tissue using 2 ml of TE-saturated phenol. After mixing, 0.5 ml of the phenol extract was added to 100 μl of chloroform and 600 μl of dH_2_O. The samples were mixed vigorously and centrifuged at 10,000xg for 5 minutes. The aqueous phase was removed and used for ATP determination at a 1:1000 dilution. A Luciferase-based ATP determination kit was used to analyze the samples (Molecular Probes A22066). Following the kit protocol, Luciferase activity was read on the Biotech Synergy H1 (Winooski, VT) plate reader with an integration time of 5 seconds. Samples were run in triplicate and compared to a standard curve to determine the quantity of ATP.

### Statistics

All data are presented as Mean±SEM. Comparison between two groups was performed with unpaired student’s t-test. A value of p<0.05 was considered significant.

## Results

The newly designed animal holder and ^1^H/^31^P coil system (Fig 1) was tested on the bench and inside the scanner. Mouse heart rate, breathing rate and temperature were measured, and no abnormal physiological changes caused by this new system were observed.

**Fig 2:**
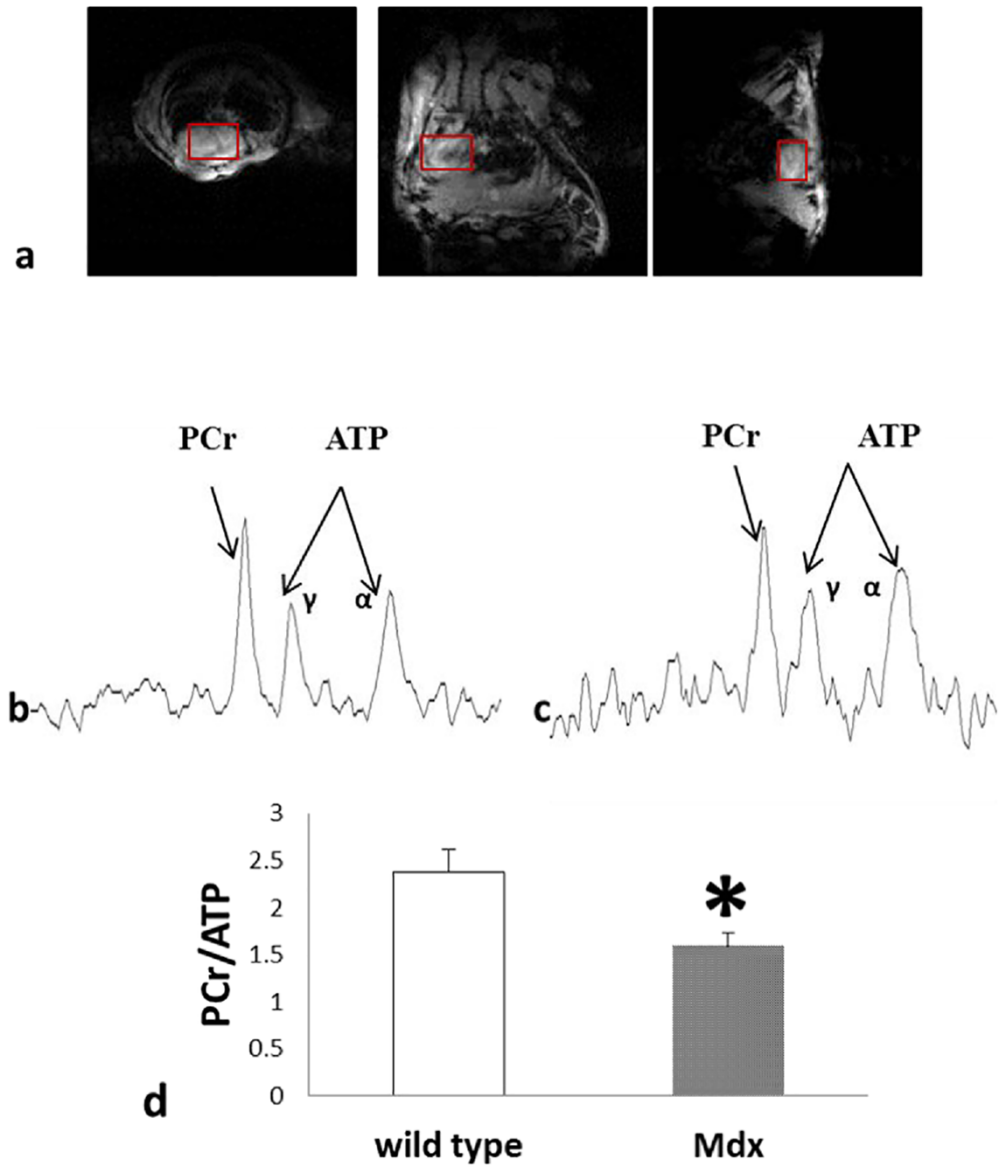
In vivo cardiac images and spectrum. Fig 2a showed 3 representative images from three dimension, axial, coronal, and sagittal. (repetition time TR = 180ms, TE = 3.04ms, flip angle 20 deg, CINE frames 2, Averages = 2, matrix = 128x128, field of view 40x40mm, slice thickness 2mm). Fig 2b and 2c were the 3D single voxel heart ^31^P spectrum of C57BL/10 mouse and mdx mouse (ISIS, TR = 4s, spectral width 10 kHz, average 768). Fig 2d showed the summarized PCr/ATP ratio (PCr/ATP = 1.59±0.13 vs 2.37±0.25. *, p<0.05, wild type: C57BL/10).

Total 15 mice were scanned for the study. ^1^H images acquired with gated tagcine sequence showed clear heart images from 3 dimensions, axial, coronal and sagittal. To ensure no contamination from adjacent tissue, the selected voxel consisted of only heart tissue on all 15 slices from axial, coronal and sagittal dimension. Without repositioning the animal, the 3D single voxel ^31^P spectrum was acquired with gated 3D-ISIS. The total acquisition was 60 min. The resonance peaks of PCr, and ATPγ were integrated. Dystrophic mdx mice showed significantly decreased PCr/ATP compared with control group C57BL/10 mice (1.59±0.13 VS. 2.37±0.25, P<0.05) (Fig 2).

**Fig 3:**
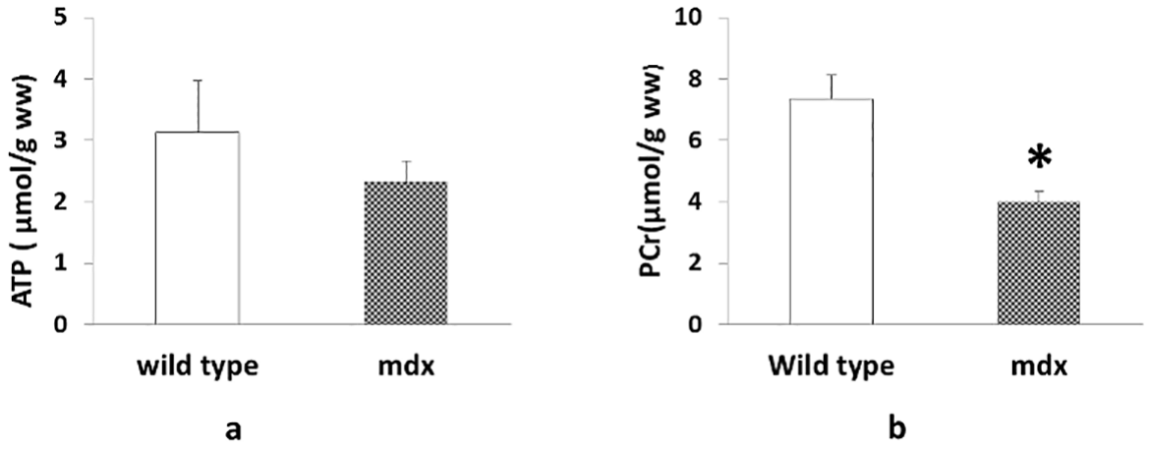
a) Myocardial ATP levels determined by bioluminescence. ATP levels for C57Bl10 (n = 3) and mdx (n = 4) mice were determined and normalized to total crushed heart tissue. No significant differences were found using Student’s T-test (p-value = 0.369). b) The calculated myocardial PCr level based on the average [ATPγ] in Fig 3 are summarized. (*, p<0.01, ww: weight weight).

**Fig 4:**
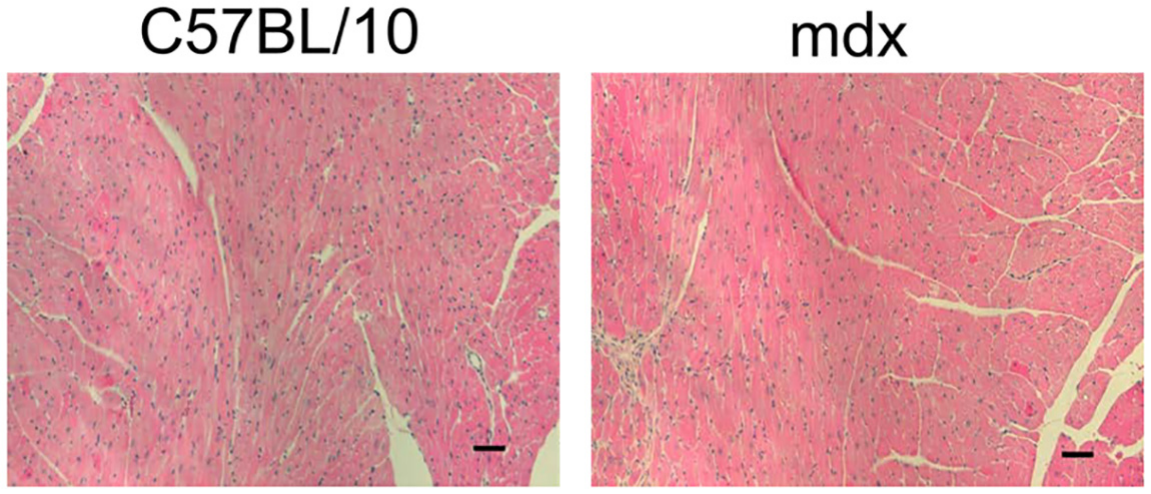
Histopathology of the heart of C57BL/10 and mdx of the same age used in these studies. Bar = 50 μm.

After the MR scanning, animals were sacrificed and hearts were rapidly explanted and frozen in liquid nitrogen for myocardial ATP concentration measurement. There was no significant difference in the ATP concentration from C57BL/10 (3.12±0.84 μmol/g wet weight) and mdx (2.32±0.34 μmol/g tissue) hearts (Fig 3a). Using this myocardial ATP concentration and the in vivo PCr/ATP ratio, the PCr concentration was determined. These studies revealed that dystrophic mice have significantly lower in vivo PCr levels compared to C57BL/10 hearts (3.69±0.37 VS. 7.41±0.76 μmol/g wet weight, p<0.05); Fig 3b). Importantly, these changes in myocardial bioenergetics are present in hearts from young dystrophic mice with little evidence of fibrosis or structural changes (Fig 4).

## Discussion

Although dystrophic hearts may appear stable for a period of time, the initial hemodynamic stability is followed by the development of LV dysfunction that leads to dilated cardiomyopathy (DCM) and heart failure. The mechanisms that contribute to the transition from the compensated state to DCM remain unclear [11]. The studies reported here indicate that the transition to failure is preceded by significant alterations in myocardial bioenergetic state that are reflected by the PCr/ATP ratio [12, 13]. Furthermore, our finding indicates that these energetic changes precede changes in cardiac structure or contractile function. Reductions in the bioenergetic state of the dystrophic heart provide a molecular mechanism for the reduced cardiac reserve observed in these hearts.

Here, we provide new data highlighting a non-invasive closed chest ^31^P MRS method using a 9.4 T magnet in control and dystrophin-deficient mdx mice (Fig 2). The novel NMR technology allows the serial assessment of the myocardial bioenergetics in the mouse. To the best of our knowledge, this study represents the first report of completely non-invasive cardiac MRS in the mdx mouse. This advance provides the first direct evidence of a significant decrease in the PCr/ATP ratio in mdx myocardium compared to dystrophin replete C57BL/10 mice at age of 5–6 months when baseline hemodynamic and LV structure are normal [7, 14]. The reduction in the PCr/ATP ratio provides important insights into the energetic state of the heart. We have previously demonstrated that in the in vivo heart the reduction of myocardial PCr/ATP is linearly related to the severity of LV dysfunction and LV hypertrophy in failing hearts [13, 15, 16]. In hearts with congestive heart failure (CHF), there is a reduction in the rate of myocardial ATP production via creatine kinase (CK) that is also linearly related to the severity of LV chamber dysfunction [17, 18].

The energetic deficits observed in this study raise several important points. First, the significant myocardial energetic deficits observed in mdx mice aged ~5–6 months. These energetic changes precede evidence of cardiomyopathy by histopathology or baseline LV hemodynamics [14, 19, 20]. The reduction in myocardial energetic state also provides insight into the potential mechanism limiting the cardiac reserve of dystrophic mice, where various forms of stress, such as beta-adrenergic stress or pressure-overload, demonstrate a cardiomyopathic phenotype in dystrophic mice [7, 21–23]. Importantly, It has been recently reported that DMD patients have reduced PCr/ATP ratios and that the severity of myocardial bioenergetic abnormality is linearly related to the severity of LV pump dysfunction [24]. The mechanisms underlying this myocardial bioenergetic deficit are not clear at present. The absence of the membrane protective function of dystrophin presents several metabolic challenges to the dystrophic heart which may increase the energy expenditure of the heart, resulting in a constant state of relative energy depletion. It is also possible that mitochondrial dysfunction resulting from the dystrophic process may result in the observed defective energetic state.

In conclusion, the present study provides the first evidence of in vivo bioenergetic deficits in the young dystrophic mouse heart whose cardiac structure and contractile function is relatively normal. This finding represents a potential highly valuable early in vivo biological signature to track disease progression in the dystrophic heart. The results also provide insight into the pathophysiological role of energetics in dystrophic cardiomyopathy. Clinical management of heart disease in DMD patients is challenging owing to the lack of high fidelity functional markers of disease. The demonstration of in vivo energetics as an early non-invasive physiological marker of emerging dystrophic cardiomyopathy could be critical for the evaluation of therapies aimed at slowing or reversing the cardiomyopathy of DMD.
